# Post-Operative Monitoring of Intestinal Tissue Oxygenation Using an Implantable Microfabricated Oxygen Sensor

**DOI:** 10.3390/mi12070810

**Published:** 2021-07-10

**Authors:** Jamie R. K. Marland, Mark E. Gray, David J. Argyle, Ian Underwood, Alan F. Murray, Mark A. Potter

**Affiliations:** 1 School of Engineering, Institute for Integrated Micro and Nano Systems, University of Edinburgh, Scottish Microelectronics Centre, King’s Buildings, Edinburgh EH9 3FF, UK; ian.underwood@ed.ac.uk; 2 The Royal (Dick) School of Veterinary Studies and Roslin Institute, University of Edinburgh, Easter Bush, Roslin, Midlothian EH25 9RG, UK; mark.gray@ed.ac.uk (M.E.G.); david.argyle@roslin.ed.ac.uk (D.J.A.); 3 School of Engineering, Institute for Bioengineering, University of Edinburgh, Faraday Building, Edinburgh EH9 3DW, UK; alan.murray@ed.ac.uk; 4 Department of Surgery, Western General Hospital, Crewe Road, Edinburgh EH4 2XU, UK; mark.potter@ed.ac.uk

**Keywords:** oxygen sensor, electrochemical, microfabricated, oxygenation, anastomotic leakage

## Abstract

Anastomotic leakage (AL) is a common and dangerous post-operative complication following intestinal resection, causing substantial morbidity and mortality. Ischaemia in the tissue surrounding the anastomosis is a major risk-factor for AL development. Continuous tissue oxygenation monitoring during the post-operative recovery period would provide early and accurate early identification of AL risk. We describe the construction and testing of a miniature implantable electrochemical oxygen sensor that addresses this need. It consisted of an array of platinum microelectrodes, microfabricated on a silicon substrate, with a poly(2-hydroxyethyl methacrylate) hydrogel membrane to protect the sensor surface. The sensor was encapsulated in a biocompatible package with a wired connection to external instrumentation. It gave a sensitive and highly linear response to variations in oxygen partial pressure in vitro, although over time its sensitivity was partially decreased by protein biofouling. Using a pre-clinical in vivo pig model, acute intestinal ischaemia was robustly and accurately detected by the sensor. Graded changes in tissue oxygenation were also measurable, with relative differences detected more accurately than absolute differences. Finally, we demonstrated its suitability for continuous monitoring of tissue oxygenation at a colorectal anastomosis over a period of at least 45 h. This study provides evidence to support the development and use of implantable electrochemical oxygen sensors for post-operative monitoring of anastomosis oxygenation.

## 1. Introduction

Creation of a surgical anastomosis (a connection between two hollow structures) following intestinal resection is generally highly successful. However, if healing of the anastomosis fails, luminal contents can leak into the abdomen causing septic peritonitis—a dangerous and often lethal complication. Overall anastomotic leak (AL) rates are typically 6–7%, but can be as high as 24% for rectal operations [1,2]. Patients with an AL have significantly increased mortality of up to 32% [3], and poorer oncological outcomes [4]. AL also has substantial associated economic costs, estimated at USD 28.6 M per thousand patients [5]. Development of new technologies for monitoring anastomosis patients to reduce the incidence of AL and improve clinical outcomes, are, therefore, a focus of substantial clinical and engineering effort [6].

Insufficient tissue oxygenation is a key physiological factor influencing anastomotic leakage. It leads to impaired wound-healing, necrosis (tissue death), and dehiscence (separation of the joined tissue) [7,8]. Multiple clinical studies have shown that intra-operative assessment of intestinal perfusion or oxygenation immediately after resection can be predictive of intestinal AL [6]. The techniques used in these studies include laser fluorescence angiography with indocyanine green, and laser Doppler flowmetry for measuring local blood flow [9,10]; near-infrared spectroscopy and visible light spectroscopy for measuring tissue haemoglobin oxygen saturation (StO_2_) [11,12]; pulse oximetry for arterial haemoglobin oxygen saturation (SpO_2_) [13]; and polarography using a Clark-type electrode to directly measure the partial pressure of O_2_ in tissue (ptO_2_) [14]. These studies have established the importance of tissue oxygenation in the aetiology of AL, and they demonstrate the value of sensor technologies in assisting clinical decision making.

However, intra-operative techniques cannot fully predict the development of AL, and many of the pathological processes associated with leakage occur post-operatively [7,8]. Suspected leaks are typically investigated using radiological imaging modalities such as computerised tomography (CT) and contrast radiography, but these are limited by cost and patient radiation exposure, and they introduce delays before AL treatment commences [6]. Continuous post-operative monitoring of local tissue oxygenation is, therefore, a highly desirable alternative. It could provide a means to assess anastomotic healing easily and rapidly, optimising patient treatment planning. For example, interventions to improve oxygenation of the anastomosis may prevent a leak from occurring, while early leak diagnosis may enable revision surgery to be performed before sepsis becomes established. In principle, post-operative monitoring could be achieved using one or more implantable oxygen sensors placed at the anastomotic site during surgery. 

Commercial oxygen sensors are typically bulky and unsuitable for implantation, so we previously developed a miniature electrochemical Clark-type tissue oxygen sensor to address this need [15]. Electrochemical oxygen sensors operate by biasing a platinum electrode at an oxygen reducing potential, with a resulting current that is proportional to oxygen partial pressure. Our previous sensor was microfabricated on a silicon die, and contained a platinum working electrode (WE) and counter electrode (CE), and an on-chip Ag/AgCl reference electrode (RE). Its surface was protected with a thin ionomer membrane. We showed that it was effective at detecting intestinal ischaemia and tissue hypoxia in a rat model [16]. However, it suffered from a short lifetime due to failure of its RE, and was susceptible to biofouling, causing a loss of sensitivity [15]. Here, we introduce an enhanced version of the sensor that addresses these problems. We describe its bench characterisation and the validation of its performance in a pig colorectal anastomosis model.

## 2. Materials and Methods

### 2.1. Electrode Fabrication

Devices were fabricated on 100 mm diameter N-type <100> silicon wafers (Si-Mat). First, a silicon dioxide layer (thickness 500 nm) was grown on the wafer surface by thermal oxidation to insulate the electrodes from the bulk silicon. The platinum electrode and interconnect layer (thickness 50 nm), with an underlying titanium adhesion layer (thickness 10 nm), were then deposited by electron beam evaporation, and patterned by a photolithography and photoresist lift-off process. The aluminium bond pad layer (thickness 1000 nm) was deposited next by sputtering, then patterned by a photolithography and photoresist lift-off process. Finally, an upper passivation layer of silicon dioxide (thickness 500 nm) was deposited by plasma-enhanced chemical vapour deposition, then patterned by photolithography and reactive ion etching using an O_2_/CF_4_ plasma to define and expose the electrode and bond pad areas (Supplementary Figure S1). A temporary protective layer of photoresist was deposited on the wafer to avoid corrosion and physical damage, followed by dicing into individual 2 mm × 3 mm dies, each containing a single microelectrode array.

### 2.2. Assembly and Packaging

A custom 1.7 mm × 33.1 mm flexible polyimide printed circuit board (PCB) (Merlin Flex Ltd., Hawarden, UK) was used for sensor assembly. The board was manufactured from two layers: a bottom layer (25 µm polyimide and 25 µm adhesive) with a single copper track (thickness 35 µm), and a top layer (also 25 µm polyimide and 25 µm adhesive) of coverlay. A stiffener (250 µm polyimide) was added to the bottom layer to improve mechanical stability in the die mounting and soldered connection areas. Exposed copper regions were created in the coverlay for wire bonding and soldering. All exposed copper was ENIG (electroless nickel immersion gold)-coated. A 50 cm biocompatible PVC-coated 30 AWG copper wire lead (Alpha Wire, Elizabeth, NJ, USA) was soldered to the flex for connectivity to external instrumentation.

The sensor die was stripped of its protective photoresist, then mounted on the flexible circuit board using Loctite 4014 biocompatible adhesive (Loctite, Westlake, OH, USA). Gold wire bonds between the flexible PCB track and die pad were produced by ultrasonic ball bonding. Encapsulation was then performed using EPO-TEK OG116-31 biocompatible UV-curable epoxy (Epoxy Technology Europe Ltd., Marlborough, UK) as previously described [15]. In brief, epoxy was deposited on the whole assembly and cured over the wire bonds and die edges by selective UV exposure through a glass photomask. Uncured epoxy was removed using acetone, producing a package of approximately 2.8 mm × 5.1 mm × 1.4 mm (W × L × H) with an open window over the electrodes of approximately 1.4 mm × 1.8 mm (W × L). Epoxy was then manually applied to the back of the sensor, its front and rear edges, and the soldered sensor lead connection, then cured by flood UV exposure. The packaged sensor was baked at 80 °C for 2 h to ensure biocompatibility of the cured epoxy, then at 150 °C for 5 min (above the epoxy glass transition temperature of 137 °C) to improve its hermeticity [17]. Sensors to be used for implantation were sterilised by exposure to ethylene oxide (AN-73 EtO capsule, Andersen Products, Clacton-On-Sea, UK) for 12 h at room temperature and pressure, then degassed in air for >2 h, prior to hydrogel casting.

### 2.3. Hydrogel Casting

Preparation of the poly(2-hydroxyethyl methacrylate) (pHEMA) sensor coating was based on a previously described protocol [18], and was performed under cleanroom conditions. All reagents were obtained from Merck Life Science UK Ltd. (Gillingham, UK). In brief, 2-hydroxyethyl methacrylate (HEMA) was pre-treated with inhibitor remover beads (306312), then filtered to remove the beads. A hydrogel precursor mixture was made containing (% *w*/*w*): 63% HEMA monomer, 1% ethylene glycol dimethacrylate crosslinker, 35% deionised water, and 1% 2-hydroxy-2-methylpropiophenone UV photo-initiator. The mixture was stirred for 10 min in darkness, then filtered. Each sensor cavity was filled with 1.5 µL of the mixture (giving a hydrogel depth of approximately 0.6 mm), then exposed to UV light for 5 min to polymerise the poly-HEMA (pHEMA) hydrogel. Finally, sensors were transferred to a large volume of sterile phosphate buffered saline (PBS), with a composition of 154 mM NaCl and 10 mM phosphate buffer at pH 7.4, for at least 1 day prior to use. This provided time to allow unreacted precursors to diffuse out of the hydrogel, and to equilibrate it with the saline electrolyte.

### 2.4. Electrochemistry

Bench characterisation was performed using an Autolab PGSTAT12 potentiostat equipped with a MUX.MULTI4 multiplexer (Metrohm AG, Herisau, Switzerland) and controlled by NOVA 1 software. Cyclic voltammetry (CV) was performed with a sweep rate of 100 mV/s and a step size of 2 mV. Chronoamperometry (CA) was performed for 20 s with a 100 ms sample interval. A double-junction Ag/AgCl electrode (Merck Life Science UK Ltd.) with a saturated KCl outer filling solution was used as a combined counter/reference electrode for all in vitro measurements. PBS was used as an electrolyte for sensor characterisation. Oxygen sensitivity was measured in solutions degassed using argon until the required oxygen partial pressure was achieved. Oxygen partial pressure in solution was measured using a Seven2Go S9 meter with an InLab OptiOx probe (Mettler Toledo, Leicester, UK). Oxygen response speed was assessed in the same degassing apparatus, using repeated measurements with an interval of 2 min. Susceptibility to biofouling was assessed in 35 mg/mL bovine serum albumin (Merck Life Science UK Ltd.) in air-saturated PBS, using repeated measurements with an interval of 60 min.

In vivo recordings and pre/post-implantation sensor testing were performed using an EmStat3 Blue portable potentiostat equipped with a MUX8-R2 multiplexer (PalmSens BV, Houten, The Netherlands) and controlled by PSTrace 5.4 software. The potentiostat was powered by a 10.8 Ah USB powerpack, and connected via Bluetooth to minimise pickup of electrical noise from the control computer. CA was performed for 20 s with a 100 ms sample interval, as used for bench characterisation. Measurements were repeated at intervals of 2 min. A BlueSensor P P-00-S electrocardiogram Ag/AgCl electrode (Ambu Ltd, Alconbury Weald, UK) placed on the abdomen skin was used as a combined counter/reference electrode for all in vivo measurements. Pre/post-implantation sensor testing was performed in sterile PBS using a double-junction Ag/AgCl electrode as a combined counter/reference electrode.

### 2.5. Animals

In vivo studies were carried out under a UK Home Office Project Licence in accordance with the Animals (Scientific Procedures) Act 1986. Approval to conduct these studies was obtained from the University of Edinburgh Animal Welfare and Ethical Review Boards. Three juvenile (2–3 months of age) female Landrace cross commercial breed pigs, each weighing 30–45 kg, were transported from the farm of origin and used immediately.

### 2.6. General Anaesthesia

Specialist veterinary anaesthetists or veterinary surgeons registered in a specialist training programme managed all aspects of anaesthesia. Details of all drug doses and manufacturers are provided within Supplementary Table S1. All pigs received intramuscular pre-anaesthetic medication consisting of morphine, medetomidine, alfaxalone and midazolam. Once adequate sedation was apparent, general anaesthesia was induced with isoflurane (1–3%) delivered in 100% O_2_ via a mask. All pigs underwent tracheal intubation with an endotracheal tube (ET). Two pigs required intravenous alfaxalone to facilitate intubation. Following inflation of the ET cuff, general anaesthesia was maintained with isoflurane, vaporised in an O_2_/air mixture (FiO_2_ 0.45–0.55) in combination with partial intravenous anaesthesia (alfaxalone, morphine, medetomidine and midazolam) given as a continuous rate infusion. Inhaled gases were provided through a Lack or circle breathing system connected to the ET with end-tidal isoflurane concentrations of 1.5–2.0% used to maintain unresponsiveness. Mechanical lung ventilation was provided to achieve a tidal volume of 8–10 mL/kg. Instrumentation consisted of surgical placement of a 7 Fr jugular venous catheter and a 3 Fr carotid arterial catheter. Intravenous fluids were provided through the jugular catheter to replace lost fluids/electrolytes, sustain cardiac preload and help maintain mean arterial blood pressure in the range of 70–80 mmHg. Arterial blood samples, taken from the carotid cannula, were used for intermittent biochemical, haematological and blood-gas analysis using a portable blood gas electrolyte and critical care analyser (epoc, Woodley Equipment Company Ltd, Bolton, UK). A multiparameter monitoring device (Datex-Ohmeda S/5, SOMA Technology, Bloomfield, CT, USA) was used to assess electrical activity of the heart, pulse rate and blood pressure, in combination with pulse oximetry, capnography, spirometry and inspired/expired gases (inhaled anaesthetic agent, CO_2_ and O_2_). Core body temperature was monitored using rectal and oesophageal thermistors and maintained in the range of 38.5–39.5 °C. Pre-emptive analgesia was provided at the time of pre-anaesthetic medication. All pigs received intravenous cefuroxime every 8 h. One pig required a single intravenous injection of neutral insulin (0.5 IU/kg), a constant rate infusion of glucose (60–500 mg/kg/h) and a single intravenous injection of frusemide (1 mg/kg) to correct hyperkalaemia, maintain normoglycemia and maintain urine output, respectively.

### 2.7. Surgery

Two surgical groups were used, consisting of either jejunal artery ligation in combination with FiO_2_ alterations, or colorectal anastomosis. The pig undergoing colorectal anastomosis received a warm water rectal enema immediately after anaesthesia induction. All animals underwent a ventral midline celiotomy. A 12 Fr Foley catheter was also placed in the bladder and connected to a closed urinary collection system. All animals were euthanised with intravenous sodium pentobarbitone following completion of the protocol. Post-mortem examination was performed to assess the extent of ischaemic pathology, assess anastomotic integrity, and recover the sensors for post-implantation analysis. Intestinal samples were also obtained for histopathology.

#### 2.7.1. Jejunal Artery Ligation

A 50 cm section of jejunum was exteriorised. Two sensors were placed in the centre of a 20 cm section of jejunum that was later rendered ischaemic, and two were placed on a control proximal segment of jejunum with a normal vascular supply. All sensors were placed on the antimesenteric border of the intestine and sutured in place with 2 M PDS II (polydioxanone, Ethicon, Raritan, NJ, USA). Sensors were kept moist and placed at the midpoint between small serosal blood vessels. Sensor leads were exteriorised through the body wall and connected to external instrumentation. Jejunal arteries supplying the 20 cm section of jejunum encompassing the test area were individually ligated with 2 M PDS II (polydioxanone, Ethicon) and sectioned to create a localised area of ischaemia. The ventral midline celiotomy was closed routinely; 3 M PDS II (polydioxanone, Ethicon) was used for *linea alba* and subcutaneous tissue layer closure and 3 M Prolene (Polypropylene, Ethicon) was used for skin closure.

#### 2.7.2. Colorectal Anastomosis

A circular double stapled colorectal resection and anastomosis was performed as previously described in detail [19]. Briefly, the colon and anterior rectum were mobilised and isolated with laparotomy pads at the site of the proposed resection and anastomosis, approximately 7 cm from the anus. At this location, the vasa recta of the inferior mesenteric artery and vein were ligated with 2 M PDS II (polydioxanone, Ethicon) and divided. A linear stapling device was placed across the upper rectum, sealing the distal end. The proximal end was transected with a scalpel and a further 5 cm of proximal colon was resected. The proximal part of anastomosis was first prepared by use of a purse-string clamp with 2 M Prolene (Polypropylene, Ethicon) on a straight needle. The anvil component of a 25 mm curved intraluminal stapling device (Endo-Surgery ILS 25, Ethicon) was placed in the intestinal lumen and the Prolene (Polypropylene, Ethicon) suture tightened around it. The handle portion of the stapling device was introduced per rectum towards the rectal transverse staple line. Once in position, the stapling device was opened to advance the point through the intestinal wall adjacent to the staple line. The anvil within the proximal intestine was then connected to the pointed portion of the stapling device within the rectum. With the colon and rectum held in proper alignment, the instrument was closed to compress the tissues upon which the staples were fired to form the anastomosis. The instrument was withdrawn from the rectum, and the head assembly was removed to check for intestinal donut completeness. An air leak test was then performed to verify anastomotic integrity. Four sensors were placed, two on each side of the anastomotic site on the antimesenteric border of the colon and anterior surface of the rectum and sutured in place with 2 M PDS II (polydioxanone, Ethicon). Sensors were kept moist and placed at the midpoint between small serosal blood vessels. No tension was noted on the colorectal anastomosis when lying in its normal anatomical position. As previously described, the sensor leads were exteriorised through the body wall and the ventral midline celiotomy was closed routinely.

### 2.8. Computed Tomography Imaging

A single-section SOMATOM Definition AS 64-slice helical CT system (Siemens Healthcare Ltd., Camberley, UK) was used to obtain contrast CT images of the descending colon and rectum. The imaging parameters of the scanner were 120 kVp, 35 mA, 3–5 mm collimation with 1 mm section thickness. All scans were performed to include the entire abdominal cavity. Positive contrast barium CT scans were performed immediately after euthanasia in the pig which underwent anastomotic surgery. To perform the contrast study, a rectal balloon catheter was positioned within the anus and the balloon inflated. Approximately 150 mL of Gastrografin (meglumine amidotrizoate, Bayer, Reading, UK) diluted 1:3 with water was injected through the catheter into the rectum and colon. CT scans were performed immediately after instillation of the contrast agent. Integrity of the anastomosis was evaluated through assessment of the staple line and leakage of contrast material outside the intestinal lumen. 

### 2.9. Histopathology

Intestinal tissue samples across ischaemic and anastomotic sites from pigs in both protocols underwent ‘Swiss rolling’ and were fixed for at least 24 h (depending on tissue thickness) in 4% formaldehyde (Genta Medical, York, UK). Tissues underwent processing and paraffin embedding before sections were cut for haematoxylin and eosin staining as previously described [20].

### 2.10. Statistical Methods

Long-term recordings were analysed using MATLAB R2020b (MathWorks, Natick, MA, USA) to produce a time-series of steady-state current (the mean of the last 15–20 s) in each CA recording. Microsoft Excel was used for all additional data handling. Statistical analysis was performed with Prism 8 (GraphPad Software, San Diego, CA, USA). Linear regression was used to calculate oxygen sensitivity and drift. A repeated measures one-way ANOVA followed by a Bonferroni post-hoc test was used to test for differences among multiple groups with a single factor (FiO_2_ absolute differences analysis), and a repeated measures two-way ANOVA followed by Bonferroni post-hoc testing was used to test for differences between multiple groups with two factors (ischaemia and biofouling analyses). A one-sample *t*-test was used to test for deviation from a hypothetical value of zero (FiO_2_ relative differences analysis). Normality of all datasets was confirmed using a Shapiro–Wilk test prior to parametric analysis. All data in text and figures are presented as mean ± standard error of the mean.

## 3. Sensor Design

**Figure 1:**
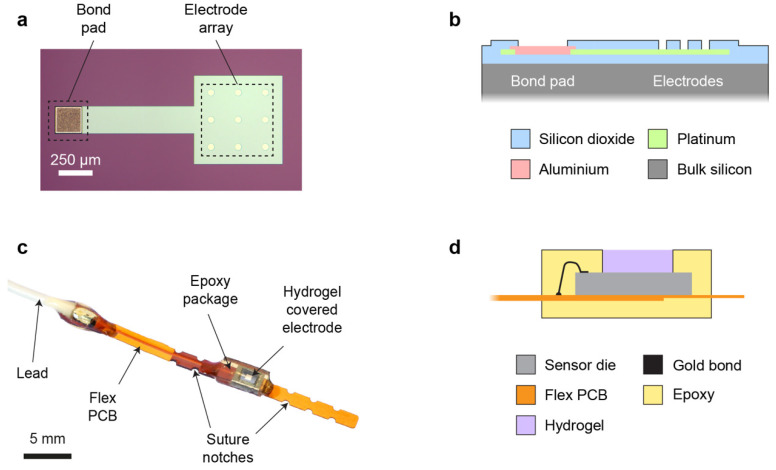
Sensor design and fabrication. (**a**): Microscope image of the 3 × 3 microelectrode WE array fabricated on a silicon substrate. The light-coloured circles in the passivation correspond to the active electrode areas. Platinum extends under all the electrodes, and it forms an interconnect track to left of the array. (**b**): Schematic cross-section of sensor die layer stack (not to scale), showing the aluminium bond pad, interconnect, and passivation windows over platinum electrodes. (**c**): Photograph of a fully packaged sensor, flexible PCB, and connection lead. (**d**): Schematic cross-section of the sensor epoxy packaging and pHEMA hydrogel membrane (not to scale). Reproduced with permission from [15].

The sensor die (2.0 mm × 3.0 mm) was microfabricated on a silicon wafer substrate, and it consisted of a 3 × 3 array of circular platinum microelectrodes (50 µm electrode diameter, 200 µm pitch) placed at the centre of the die (Figure 1a). This array design was preferred over a single electrode to provide an increased WE current, while retaining favourable microelectrode properties, including a small diffusion layer and a steady state during chronoamperometric (CA) measurements. Since temporary post-operative monitoring applications allow the use of a wired sensor, an external off-chip Ag/AgCl electrode was used as a combined reference/counter electrode (RE/CE). The platinum WE metal layer extended under the whole array, with individual active electrode areas defined by windows in an upper passivation layer. Platinum was also used for an interconnect track (width 200 µm) between the electrode array and an aluminium bond pad at the edge of the die (Figure 1b). An overlap of 10 µm was used at the pad periphery to create a robust electrical connection between the interconnect and bond pad.

The electrode die was mounted on a flexible PCB and wire bonded to a gold-coated track on the PCB. An insulated lead was also attached to the track for connection to external potentiostat instrumentation. To allow accurate surgical placement, notches were included on the flexible PCB to enable it to be sutured securely onto tissue (Figure 1c). The sensor and its connections were encapsulated selectively in biocompatible photocurable epoxy to provide insulation and mechanical stability. A small window in the epoxy was left open over the die, creating a shallow cavity into which a thick (~600 µm) pHEMA hydrogel layer was cast (Figure 1d). This hydrogel was used to explore the effectiveness of a thick membrane for mitigating the effects of biofouling. The aim was to displace the critical tissue/sensor interface away from the WE (providing a large non-fouled internal volume of hydrogel in which to make transient oxygen measurements), and to create a large surface area through which oxygen could diffuse from tissue into the sensor (reducing the effect of biofouling on oxygen mass transport).

## 4. Results

### 4.1. Sensor Characterisation

**Figure 2:**
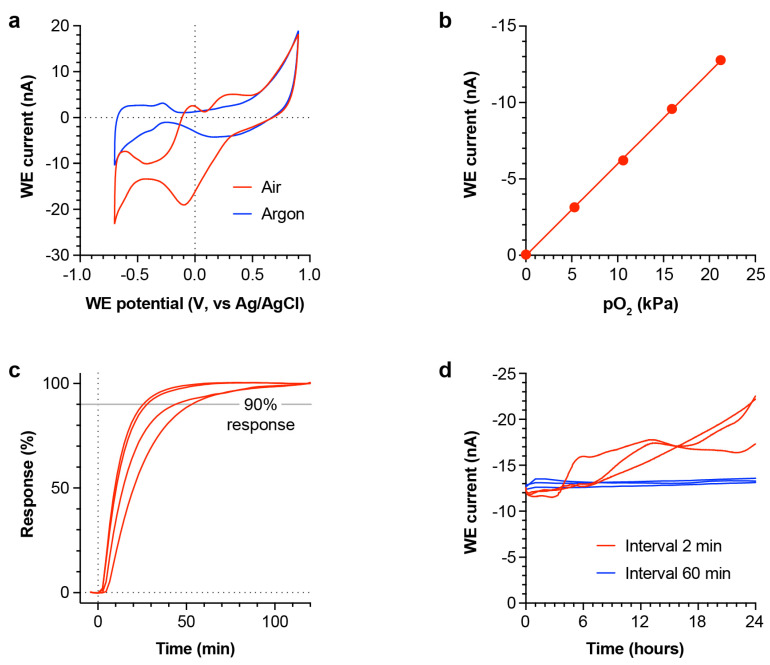
Bench characterisation of sensor performance. (**a**): Typical CV scans of sensor in PBS saturated with air (red) and PBS degassed by sparging with argon (blue). Similar results were obtained from four sensors. (**b**): Mean steady-state WE current at −0.5 V measured over a range of oxygen partial pressures in PBS (*n* = 3 sensors). (**c**): Sensor response when moved at *t* = 0 min from argon-sparged PBS (negligible oxygen) to an air-saturated PBS solution. Steady-state currents were normalised between 0% and 100% response for comparison. Grey line at 90% response indicates the threshold for determining response time. Individual responses from four sensors are shown (red lines). (**d**): Mean steady-state current at −0.5 V in air-saturated PBS over 24 h. Sensor recordings were performed at intervals of either 2 min (red) or 60 min (blue) (*n* = 3 sensors/condition).

To confirm the ability of the sensor to electrochemically detect oxygen, it was immersed in PBS and subjected to cyclic voltammetry. The expected oxygen wave was present at reducing potentials in air-saturated PBS, and it was lost following removal of oxygen from the solution by sparging with argon (Figure 2a). This showed that the sensor was effective at reducing oxygen at its WE surface. To quantify the oxygen reduction current, we selected chronoamperometry (CA) at a reducing potential of −0.5 V (vs. Ag/AgCl), as previously reported [15]. The sensor output was quantified as an average current over 15–20 s following the potential step, since a steady-state was well established by this timepoint (Supplementary Figure S2).

Oxygen sensitivity was measured in PBS at a range of oxygen partial pressure values between 0.0 kPa and 21.2 kPa (air at atmospheric pressure). The CA steady-state current was recorded at each partial pressure after allowing the sensor to equilibrate for ≥1 h. A highly linear response (R^2^ > 0.99) was observed, with a sensitivity of −0.600 ± 0.006 nA/kPa and an offset of −0.019 ± 0.076 nA (Figure 2b). Sensor response speed to a step-change in pO_2_ was measured by making repeated CA measurements following transfer of the sensor from argon-sparged (oxygen depleted) PBS to air-saturated PBS. For this analysis, each sensor output was normalised between the 0% (argon baseline) response and the 100% (air-saturated plateau) response (Figure 2c), giving a mean t_90%_ (time to reach 90% of the final response) of 37 ± 7 min (*n* = 4 sensors).

The sensor output stability was assessed by making repeated measurements in air-saturated PBS, at measurement intervals of either 2 min or 60 min (Figure 2d). Over a 24 h period, sensors measured at 2 min intervals showed a mean drift of −0.343 ± 0.070 nA/h, while sensors measured at 60 min intervals showed a mean drift of −0.017 ± 0.008 nA/h. Since the oxygen reduction current was also negative, these negative drift values indicated a slowly increasing current over time. After 24 h, the coefficient of variance (CoV) between sensor outputs was 14.1% (2 min interval) and 1.8% (60 min interval). Together, this indicates that the sensor output is more stable when longer intervals are used between successive measurements.

### 4.2. In Vivo Performance

Performance of the sensor in vivo was assessed using an anaesthetised pig model. There is substantial similarity in size and anatomy between the human and porcine gastrointestinal system [19], making it a highly relevant translational model for preclinical validation of implantable sensors. Two study designs were used: the first to test performance of the sensor, the second to investigate its suitability for intestinal anastomosis monitoring. For in vivo testing, an Ag/AgCl electrocardiogram (ECG) electrode attached to the skin was used as a combined RE/CE. ECG electrodes are specified by standard ANSI/AAMI EC12:2000 to maintain a stable electrochemical potential even while passing small continuous DC test currents (≥200 nA, for ≥8 h) or after a rapid charge transfer (≥2 mC) during defibrillation. They were, therefore, expected to also be sufficiently stable for use as a combined RE/CE in these low-current amperometric measurements. 

**Figure 3:**
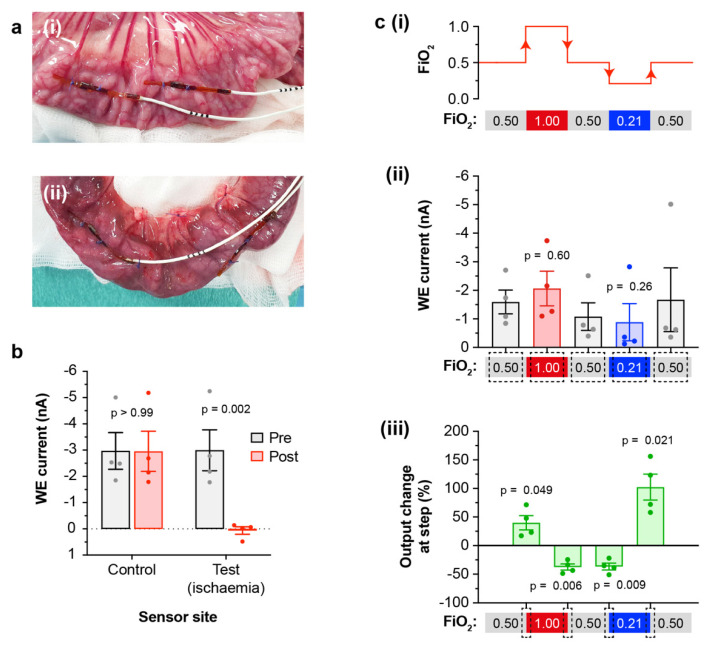
Ischaemia and FiO_2_ challenges. (**a**): Sensors fixed on intestine serosal surface at (**i**) the control site, and (**ii**) the test site (shown after it had been made ischaemic by ligation of the jejunal arteries). Similar placement was used for both pigs. (**b**): Ischaemic challenge. Mean steady-state current shown at the control and test sites, before (grey) and after (red) the test site was made ischaemic (*n* = 4 sensors/location, from two pigs). Readings were averaged over the 10 min before the ischaemia surgery, and the 10 min after. (**c**): FiO_2_ challenges. Results shown only from sensors on control (non-ischaemic) site (*n* = 4 sensors, from two pigs). (**i**) Schematic showing FiO_2_ changes in each block. (**ii**) Absolute mean steady-state current within each FiO_2_ block, averaged over the final 30 min of the block. (**iii**) Change in mean steady-state current between FiO_2_ blocks, calculated using values averaged over the final 10 min of the first block, and over the initial 10–20 min of the second block (providing 10 min for tissue oxygenation to stabilise following the change).

In the first study (pigs A1 and A2), four sensors were implanted on the small intestine to test their ability to detect ischaemia and tissue hypoxia. Of these, two sensors were implanted on a control site, and two sensors on a test site that was later rendered ischaemic. The sensors were anchored on the serosal (outer) surface using sutures over the flexible PCB notches (Figure 3a(i)), and the abdomen was closed during recordings. Later post-mortem inspection showed that the sutures provided reliable fixation. A further sensor was left free in the peritoneal cavity with the intention of measuring oxygenation of the free peritoneal fluid. Using this model, we first investigated detection of ischaemia. Initially, sensors on both the (pre-ischaemic) test and control sites showed similar outputs, as expected (Figure 3b). The test site was then rendered ischaemic by ligation of the jejunal artery branches and division of the intestine from the mesentery (Figure 3a(ii)). Following this surgery, the sensor output at the test site showed a pronounced and highly significant decrease to near-zero output, indicating clear detection of ischaemia (Figure 3b). The control site (non-operated) showed no significant change between the two measurement periods. Presence of local ischaemia at the test site was confirmed by post-mortem histological analysis, which showed loss of enterocytes and villi and haemorrhage within the submucosal layer (Supplementary Figure S3).

Using the same pigs, we then manipulated the fraction of inspired oxygen (FiO_2_) to investigate whether more subtle changes in tissue oxygenation could be detected. First, a baseline recording was made at an FiO_2_ of 0.50 (50% oxygen). Delivery of oxygen was then sequentially increased to give an FiO_2_ of 1.00, returned to 0.50, then reduced to 0.21, before finally returning to 0.50 (Figure 3c(i)). Effectiveness of these FiO_2_ interventions was confirmed by changes in central arterial blood pO_2_ (Supplementary Figure S4). Continuous sensor recordings were made throughout (Supplementary Figure S5). Analysis of the absolute sensor output at each FiO_2_ from control (non-ischaemic) sites showed that, when compared to baseline, weak trends were observed towards a higher output at FiO_2_ 1.00 and a lower output at FiO_2_ 0.21 (Figure 3c(ii)). These differences did not reach significance, as the sample size was small and there was variability between individual sensor sites. We also examined the relative differences in output from individual sensors between each FiO_2_ block. This analysis did show significant changes, appropriately positive and negative, at every FiO_2_ step (Figure 3c(iii)). Together these comparisons suggest that quantification of relative differences is likely to be more reliable than use of absolute values for patient monitoring. As expected, sensors at the ischaemic test site gave consistently very low readings throughout (Supplementary Figure S6a), while the free peritoneal sensor gave readings that mirrored those of the intestinal control site (Supplementary Figure S6b).

**Figure 4:**
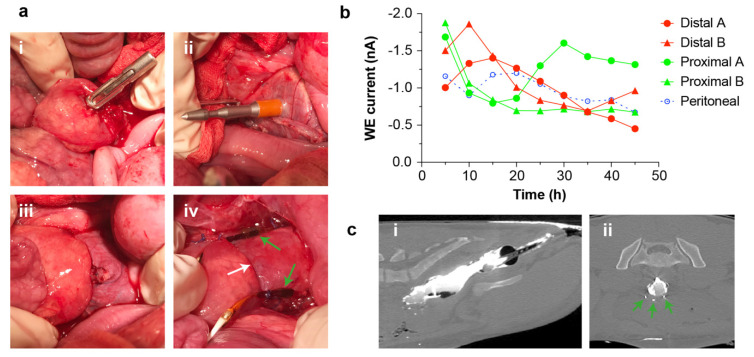
Continuous colorectal anastomosis monitoring. (**a**): Intra-operative photographs showing creation of a stapled colorectal anastomosis and sensor placement. (**i**) Proximal colon with stapler anvil secured by a purse-string suture; (**ii**) distal colon with transverse staple line and stapler spike protruding at the midpoint; (**iii**) proximal and distal colon joined by closure and firing of the stapler; (**iv**) placement of distal sensors (green arrows) at the anastomosis (white arrow). (**b**): Mean steady-state current from each sensor during the 45 h experiment, averaged over 5 h periods (five sensors, from one pig). (**c**): Post-mortem positive contrast CT images taken following a colorectal resection and anastomosis. (**i**) Transverse view. The positive contrast agent (Gastrografin) can be seen filling the rectum and distal colon. The contrast agent ran past the anastomotic site with no evidence of leakage of contrast material outside the intestinal lumen. (**ii**) Sagittal view. This image was taken through the anastomotic site. Three sensors can be identified surrounding the colon (green arrows). There is no evidence of leakage of contrast material outside the intestinal lumen.

For the second study (pig A3), we investigated suitability of the sensor for long-term monitoring of tissue adjacent to a colorectal anastomosis. A short section of colon was resected, then re-joined using a stapled anastomosis created with a circular staple gun (Figure 4a). Two sensors were placed proximal to the anastomosis, and two sensors distal to it. The sensors were fixed on the tissue using sutures, which was also found to provide sufficient security at this site. As before, an additional sensor was left free in the abdomen to measure peritoneal fluid. Sensor recordings were made continuously to monitor tissue oxygenation over 45 h. Since long-term trends are often the most informative for high dependency unit and intensive care monitoring, we averaged the sensor output over 5 h intervals. All sensors gave outputs consistent with non-ischaemic tissue, indicating good perfusion of the anastomosis (Figure 4b). No clear trends indicating the presence of tissue hypoxia were observed at either the proximal or distal sensor sites during the 45 h post-operative recording period, suggesting that the anastomosis remained well perfused by oxygenated blood throughout. This was corroborated by post-mortem positive contrast CT, which showed the anastomosis had not developed a leak (Figure 4c), and by histological examination which showed healthy tissue both proximal and distal to the anastomosis (Supplementary Figure S7). All sensors were still functioning at the end of the experiment, showing an in vivo sensor lifetime of at least 45 h. The experiment was terminated after 45 h as it was an initial proof-of-concept trial in an anaesthetised animal, and future work will explore longer monitoring periods in animals recovered from anaesthesia.

### 4.3. Assessment of Biofouling

**Figure 5:**
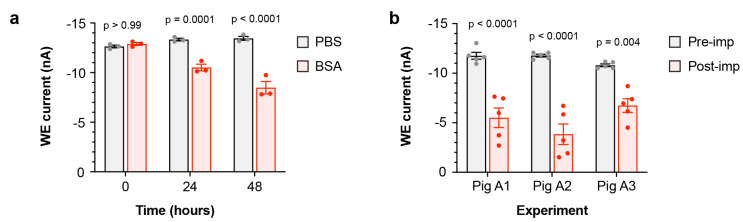
Effect of biofouling on sensor sensitivity. (**a**): In vitro assessment, showing mean steady-state WE current at 0, 24, and 48 h from sensors continuously immersed in either air-saturated PBS alone or air-saturated PBS with 35 mg/mL BSA (*n* = 3 sensors/condition). (**b**): In vivo assessment, showing mean steady-state WE current from sensors in air-saturated PBS before and after implantation in pigs A1–A3 (*n* = 5 sensors/pig).

To investigate the effect of biofouling on the O_2_ sensor in vitro, we used bovine serum albumin (BSA), a protein commonly used to model biofouling and one of the main components of peritoneal fluid [21]. Sensors were incubated in either PBS containing BSA, or PBS alone as a control, and the steady-state current output in these air-saturated solutions was measured over 48 h. A progressive and significant decline in output current was observed from sensors incubated with BSA, when compared to the control sensors in PBS (Figure 5a). This indicates that the sensor is susceptible to loss of sensitivity due to protein biofouling in vitro. We also assessed the in vivo impact of biofouling by comparing the sensor current output in air-saturated PBS before implantation and after explantation at post-mortem. A significant decrease in the sensor current was observed between the pre- and post-implantation outputs from sensors used in all in vivo studies (Figure 5b), confirming that biofouling during implantation also led to a partial loss of oxygen sensitivity.

## 5. Discussion

We demonstrated an implantable electrochemical oxygen sensor that monitors post-operative tissue oxygenation. Its key components were a platinum microelectrode array and a pHEMA membrane to mitigate the impact of biofouling. The sensor was microfabricated on a silicon substrate, allowing future integration with smart on-chip electronics to reduce dependence on external instrumentation. An external Ag/AgCl ECG electrode was used as a combined CE/RE. The miniature sensor was encapsulated in a biocompatible package to allow surgical implantation and continuous monitoring of tissue oxygenation.

We showed that the sensor had a highly linear response to oxygen in vitro. The use of a microelectrode array design increased (relative to a single electrode) the overall current output to compensate for the lower diffusion coefficient of oxygen in pHEMA (typically 1 × 10^−6^ cm^2^s^−1^ [22]) compared to water (typically 2 × 10^−5^ cm^2^s^−1^ [23]). Overall, this resulted in a similar oxygen sensitivity to our previous sensor design [15]. The lower diffusion coefficient also led to a relatively slow response time of approximately 37 min. This is unlikely to be a limitation in clinical use, as the physiological processes associated with anastomotic ischaemia and leakage take place over several hours or days [7,8]. During in vitro testing, we also identified an unexpected dependence of sensor output stability on the inter-measurement interval, with more frequent measurements leading to decreased stability. This may be due to reactive intermediates in the oxygen reduction reaction at the WE affecting the pHEMA polymer properties [24], but further work will be required to identify the cause conclusively. Since long-term trends in anastomosis oxygenation are most clinically relevant, measurement frequency can be minimised to ameliorate this effect.

Biofouling of implanted sensor membranes due to non-specific protein adsorption is a well-known phenomenon, causing loss of sensitivity by disrupting the diffusion of analytes [25]. Our use of a thick pHEMA membrane aimed to mitigate this effect by physically spacing the electrode sufficiently far from the biofouled interface to prevent it from affecting the output during a short measurement. For example, at the end of a 20 s recording, the diffusion length (approximated by $\sqrt{2Dt}$) would be expected to reach approximately 63 µm from the electrode surface. This remains well within the ~600 µm thick pHEMA hydrogel, never reaching or being affected by the outer biofouled layer. Oxygen removed from the hydrogel by reduction at the WE was then replenished slowly by diffusion through the biofouled interface during the interval between measurements. Using incubation with BSA as an in vitro protein biofouling model, we found that the pHEMA membrane allowed the sensor to retain 79% of its sensitivity after 24 h. In contrast, our previous O_2_ sensor with a thin (0.5 µm) Nafion polymer membrane, analysed using the same BSA assay, retained only 17% of its sensitivity after 24 h [15]. This is consistent with our expectation of a correlation between membrane thickness and biofouling susceptibility. Nevertheless, the thick pHEMA membrane was still affected by both BSA and the in vivo environment. This may be due to slow infiltration of proteins or smaller peptides into the pHEMA hydrogel network, or passivation of the WE surface [25]. This will be the main target for future improvement.

Sensor lifetime was ~23 h in our previous study [15], limited by failure of the on-chip thin-film Ag/AgCl RE (originally designed for wireless applications [26]). In this study, sensor lifetime was substantially improved to at least 45 h by use of an external Ag/AgCl ECG electrode. This provides confidence that the sensor will, with further refinement, be suitable for post-operative monitoring over several days during patient recovery [6]. Clearly, such an external electrode is only viable for wired sensors, but where a wired approach is possible it will eliminate the limitations of microfabricated reference electrodes [27]. The present study also validates use of an Ag/AgCl ECG electrode for low-current amperometric measurements, establishing it as a valuable experimental tool for early testing of research sensors. It may be less suitable for sensitive potentiometric measurements where millivolt differences in RE potential are significant, as the ECG electrode internal electrolyte composition is not perfectly fixed.

We developed an in vivo pig model to test the sensor, informed by our previous work in rat intestine [16]. Using this new model, we showed that the sensor was highly effective for detection of intestinal ischaemia created by arterial ligation. It was also partially effective for detecting smaller differences in tissue oxygenation caused by experimentally induced hypoxaemia and hyperoxaemia. Variability between sensors meant that relative changes in oxygenation were detected more reliably than absolute differences. This may be due to anatomical differences in sensor location, for example proximity to larger blood vessels, causing the output from each to reflect its unique tissue microenvironment. We also successfully demonstrated continuous monitoring of tissue oxygenation at a colorectal anastomosis. These proof-of-concept studies show that electrochemical detection of intestinal tissue oxygenation using this sensor architecture is suitable for translation into clinical practice. The electrochemical measurement gives an output that is directly proportional to oxygen partial pressure. Therefore, it compares favourably to near-infrared spectroscopy and visible light spectroscopy, which use StO_2_ as a non-linear proxy for oxygenation, and to pulse oximetry and laser Doppler flowmetry which require pulsatile blood flow [28].

Our future work will focus on improving the resistance of the sensor to biofouling, enabling more accurate long-term measurements to be conducted. This platform also offers the opportunity for further integration with other sensors. For example, previous studies have shown that post-operative monitoring of multiple physiological parameters downstream of ischaemia hold promise for predicting AL. These include pH [29] and lactate [30], as well as inflammatory markers such as C-reactive protein [31]. Ultimately, clinical translation of implantable oxygen sensor technology will enable monitoring for threshold values of oxygen that presage leakage, allowing timely and evidence-driven intervention. We believe that such continuous post-operative monitoring of anastomosis healing will hold a key to improving the morbidity and mortality associated with AL.

## Data Availability

The data presented in this study are available in the paper and Supplementary Materials.

## Supplementary Materials

The following are available online at https://www.mdpi.com/article/10.3390/mi12070810/s1, Figure S1: electrode array microfabrication process, Figure S2: chronoamperometry recording in PBS, Figure S3: histological appearance of normal and ischaemic jejunal segments, Figure S4: effectiveness of FiO_2_ alterations, Figure S5: real-time sensor output, Figure S6: FiO_2_ challenges at ischaemic and peritoneal sites, Figure S7: histological appearance of colorectal anastomosis site, Table S1: drugs used to provide anaesthesia and analgesia.
