# Supplementary material for: Post-Operative Monitoring of Intestinal Tissue Oxygenation Using an Implantable Microfabricated Oxygen Sensor
Source: Micromachines (Basel). 2021 Jul 10;12(7):810. doi: 10.3390/mi12070810 (PMC8303214; doi:10.3390/mi12070810)
Supplement: Supplementary file 1 [file micromachines-12-00810-s001.zip › micromachines-1293901-supplementary.pdf]

SUPPLEMENTARY MATERIAL

Post-operative monitoring of intestinal tissue oxygenation using an implantable microfabricated oxygen sensor

Jamie R. K. Marland[1,*], Mark E. Gray[2], David J. Argyle[2], Ian Underwood[1], Alan F. Murray[3], Mark A. Potter[4]

[1] School of Engineering, Institute for Integrated Micro and Nano Systems, University of Edinburgh, Scottish Microelectronics Centre, King's Buildings, Edinburgh, EH9 3FF, UK

[2] The Royal (Dick) School of Veterinary Studies and Roslin Institute, University of Edinburgh, Easter Bush, Roslin, Midlothian EH25 9RG, UK

[3] School of Engineering, Institute for Bioengineering, University of Edinburgh, Faraday Building, Edinburgh EH9 3DW, UK

[4] Department of Surgery, Western General Hospital, Crewe Road, Edinburgh EH4 2XU, UK

* Corresponding author. Email address: jamie.marland@ed.ac.uk (J. R. K. Marland)

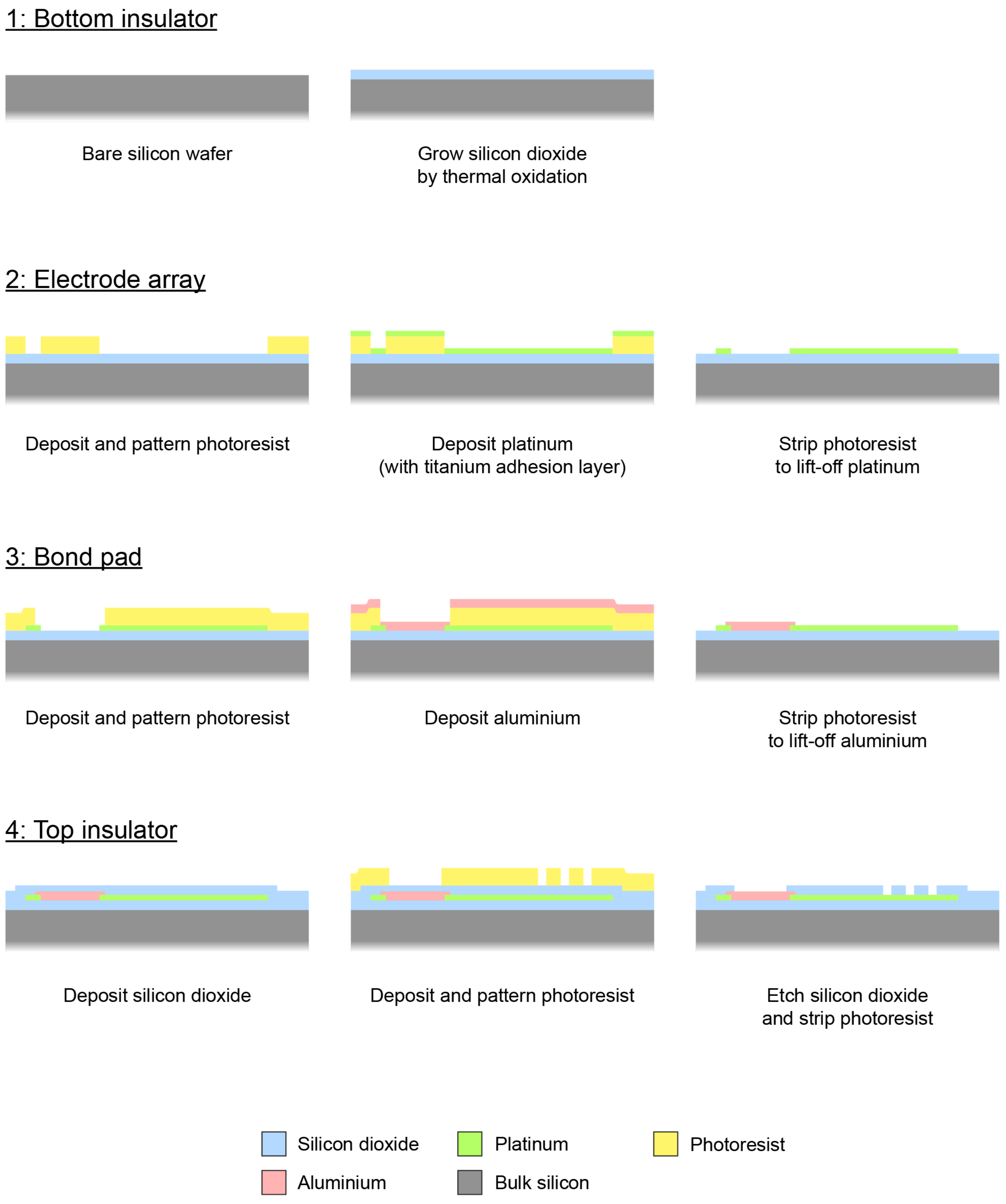

Supplementary Figure S1: Electrode array microfabrication process. Schematic cross-section of sensor die microfabrication process steps (not to scale).

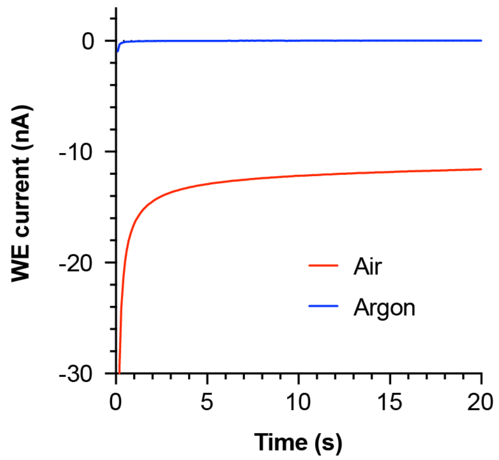

Supplementary Figure S2: Chronoamperometry recording in PBS. Typical sensor current output following a step to -0.5 V (vs external Ag|AgCl|KCl (3 M) reference electrode) in either air-saturated PBS (red line) or argon-sparged PBS (blue line). Similar results were obtained from four sensors.

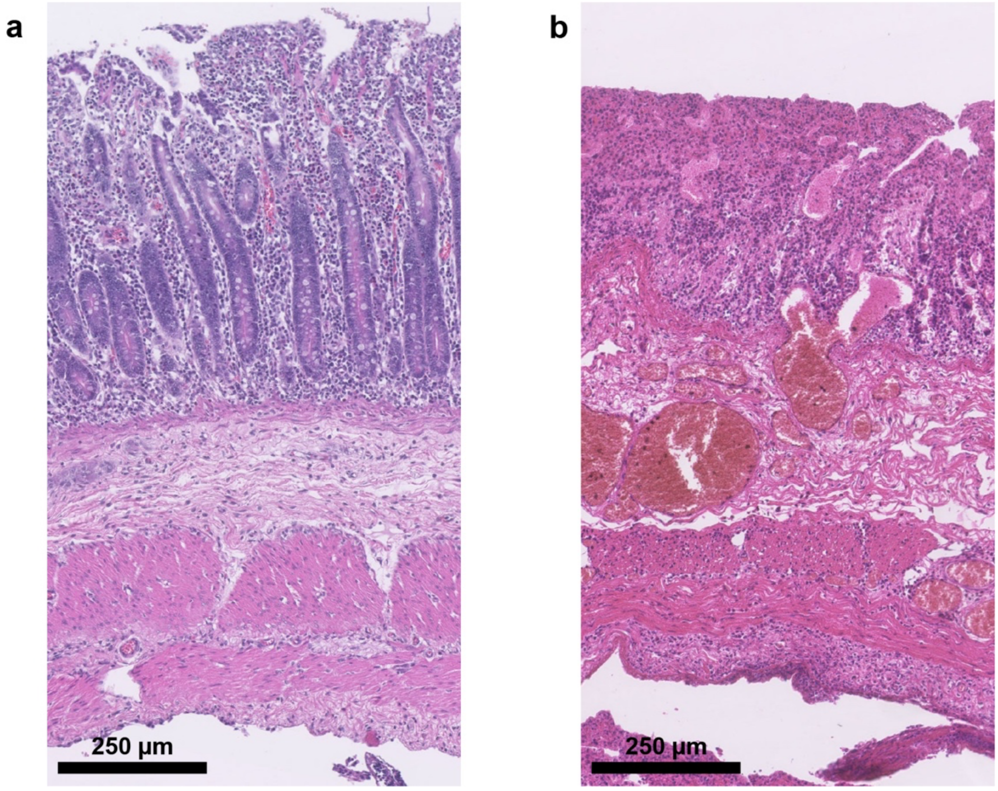

Supplementary Figure S3: Histological appearance of normal and ischaemic jejunal segments. Both microscope images are from jejunal sections stained with haematoxylin and eosin. **a:** Normal jejunum. The mucosa, submucosa, muscularis externa and adventitia are clearly demarcated as distinct layers. Enterocytes can be identified lining villi in the mucosal layer. **b:** Ischaemic jejunum. The mucosal layer is reduced in thickness with the loss of enterocytes and villi. Large numbers of erythrocytes can be seen within the submucosal layer. The adventitia appears thickened.

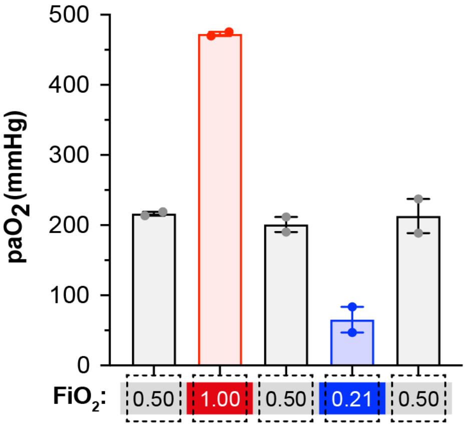

Supplementary Figure S4: Effectiveness of FiO_2 alterations. Mean arterial blood oxygen partial pressure (paO_2) measurements from Pigs A1 & A2 during the FiO_2 challenges, taken 10 min before the end of each 60 min FiO_2 block (n = 2 animals/condition).

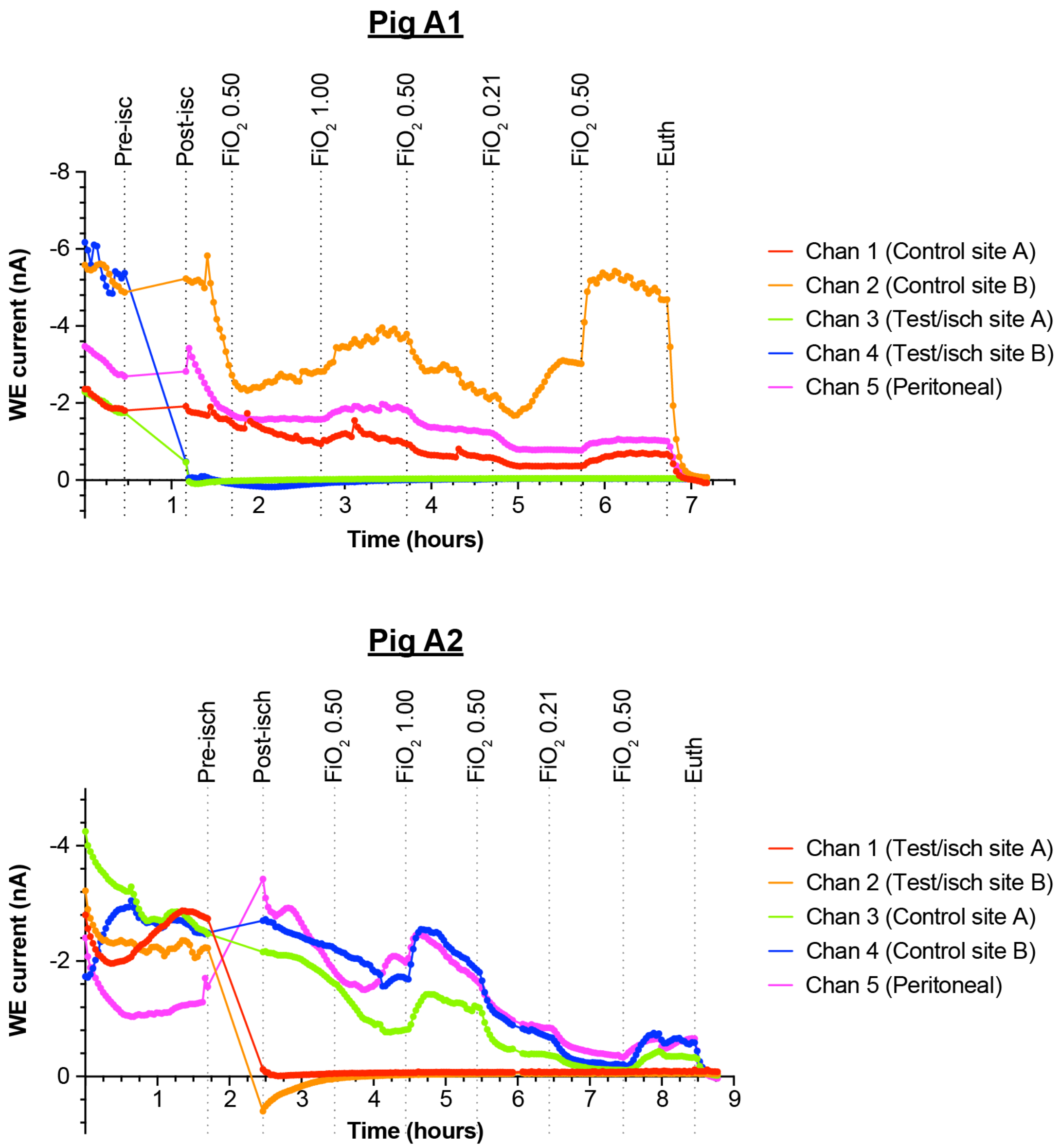

Supplementary Figure S5: Real-time sensor output. Steady-state current output from all sensors implanted in Pigs A1 & A2 at control (non-ischaemic), test (ischaemic), and peritoneal sites. Changes in output are shown during baseline stabilisation, pre/post ischaemia surgery, during manipulation of FiO_2, and following euthanasia (Euth). Recording was paused during ischaemia surgery; connecting lines are drawn between the last measurement before and the first measurement after surgery.

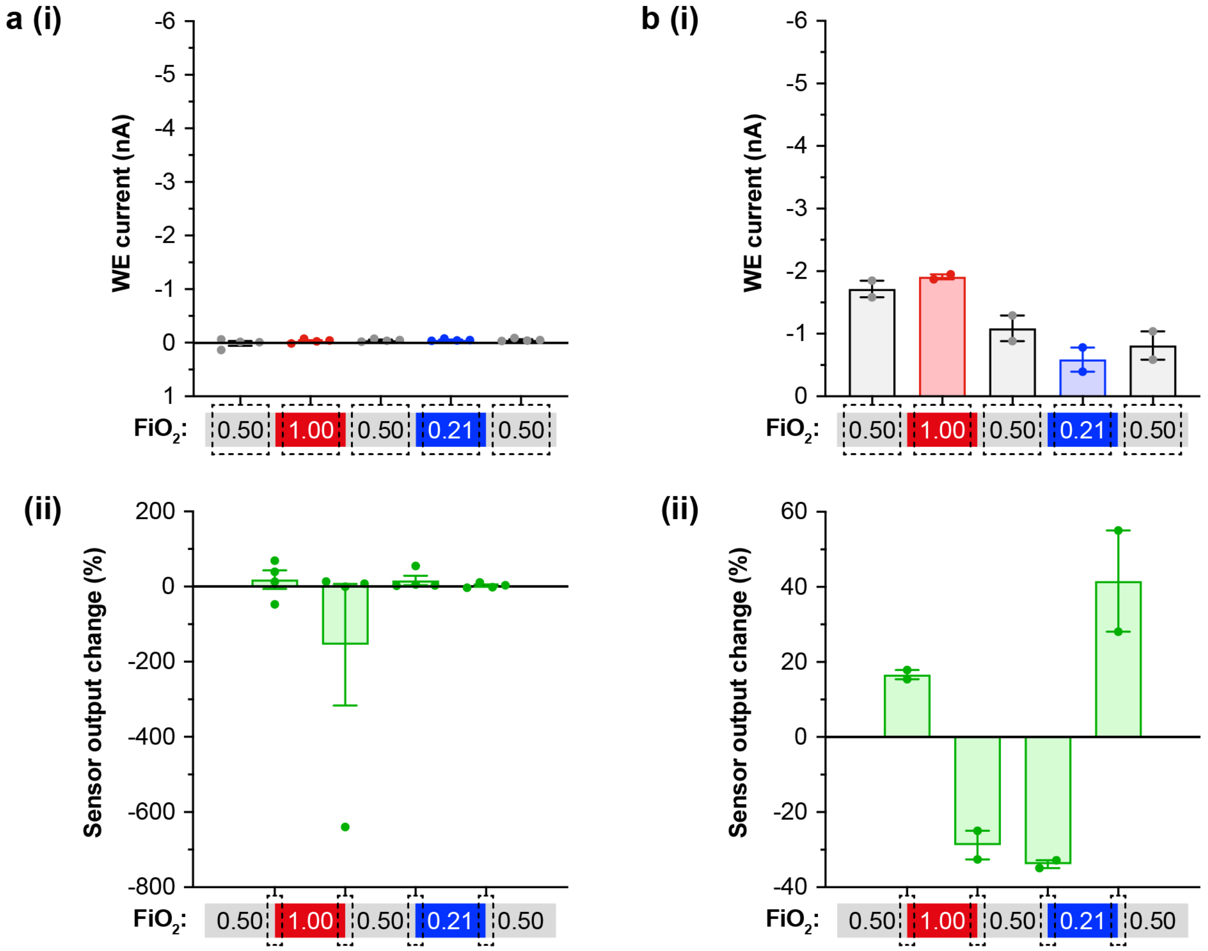

Supplementary Figure S6: FiO_2 challenges at ischaemic and peritoneal sites. a: Results from sensors on ischaemic site (n = 4 sensors, from two pigs). **b:** Results from sensors at the free peritoneal site (n = 2 sensors, from two pigs). (i) Absolute mean steady-state WE current within each FiO_2 block, averaged over the final 30 min of the block; (ii) relative change in mean steady-state WE current between FiO_2 blocks, calculated using values averaged over the final 10 min of the first block, and over the initial 10 – 20 min of the second block (providing 10 min for tissue oxygenation to stabilise following the change).

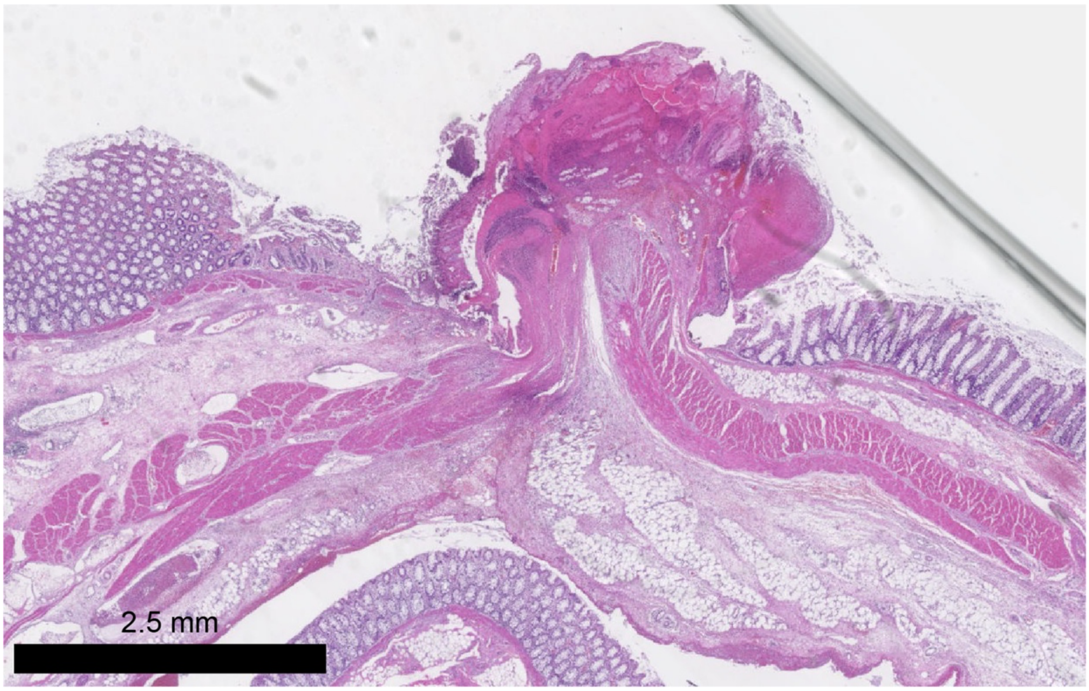

Supplementary Figure S7: Histological appearance of colorectal anastomosis site. Microscope image from the anastomosis site, stained with haematoxylin and eosin. The anastomosis site is identified by an outpouching area of muscularis into the lumen of the intestine. The mucosa, submucosa, muscularis externa and adventitia are clearly demarcated as distinct layers. Enterocytes can be identified lining villi in the mucosal layer up to the anastomotic site.

Phase	Drug	Manufacturer	Dose (mg/kg)	Route
**Sedation**	Medetomidine	'Sedator'; Dechra Veterinary Products, Shrewsbury, UK	0.007	i.m.
	Morphine	'Morphine Sulphate'; Martindale Pharmaceuticals, UK	0.3	i.m.
	Alfaxalone	'Alfaxan'; Jurox, West Sussex, UK	2	i.m.
	Midazolam	'Hypnovel'; Roche, Welwyn Garden City, UK	0.5	i.m.
**Induction**	Isoflurane	'IsoFlo'; Abbot Animal Health, Maidenhead, UK	1-3%	inhaled
	Alfaxalone	'Alfaxan' Jurox, West Sussex, UK	2	i.v.
**Maintenance**	Isoflurane	'IsoFlo'; Abbot Animal Health, Maidenhead, UK	1-3%	inhaled
	Alfaxalone	'Alfaxan'; Jurox, West Sussex, UK	0.5mg/kg/h	i.v.
	Morphine	'Morphine Sulphate'; Martindale Pharmaceuticals, UK	0.2mg/kg/h	i.v.
	Medetomidine	'Sedator'; Dechra Veterinary Products, Shrewsbury, UK	0.0025mg/kg/h	i.v.
	Midazolam	'Hypnovel'; Roche, Welwyn Garden City, UK	0.2mg/kg/h	i.v.
**Analgesia**	Morphine	'Morphine Sulphate'; Martindale Pharmaceuticals, UK	0.1-0.3	i.v./i.m.
**Intravenous fluid therapy**	Compound sodium lactate	Aqupharm No. 11, Animalcare, York, UK	5-20/ml/kg/h	i.v.
**Antibiotics**	Cefuroxime	'Zinacef'; GlaxoSmithKline, UK	20	i.v.
**Euthanasia**	Pentobarbital	'Pentoject'; Animalcare, York, UK	40	i.v.

(i.m., intramuscular; i.v., intravenous).

Supplementary Table S1: Drugs used to provide anaesthesia and analgesia.
